# Initiating maize pre-breeding programs using genomic selection to harness polygenic variation from landrace populations

**DOI:** 10.1186/s12864-015-2345-z

**Published:** 2016-01-05

**Authors:** Gregor Gorjanc, Janez Jenko, Sarah J. Hearne, John M. Hickey

**Affiliations:** Biotechnical Faculty, University of Ljubljana, 1000 Ljubljana, Slovenia; The Roslin Institute and Royal (Dick) School of Veterinary Studies, The University of Edinburgh, Easter Bush, Midlothian, Scotland UK; Agricultural Institute of Slovenia, 1000 Ljubljana, Slovenia; Genetic Resources Program, International Maize and Wheat Improvement Center (CIMMYT), Apdo, 06600 México, D.F. México

**Keywords:** Maize, Landrace, Diversity, Pre-breeding, Genomic selection

## Abstract

**Background:**

The limited genetic diversity of elite maize germplasms raises concerns about the potential to breed for new challenges. Initiatives have been formed over the years to identify and utilize useful diversity from landraces to overcome this issue. The aim of this study was to evaluate the proposed designs to initiate a pre-breeding program within the Seeds of Discovery (SeeD) initiative with emphasis on harnessing polygenic variation from landraces using genomic selection. We evaluated these designs with stochastic simulation to provide decision support about the effect of several design factors on the quality of resulting (pre-bridging) germplasm. The evaluated design factors were: i) the approach to initiate a pre-breeding program from the selected landraces, doubled haploids of the selected landraces, or testcrosses of the elite hybrid and selected landraces, ii) the genetic parameters of landraces and phenotypes, and iii) logistical factors related to the size and management of a pre-breeding program.

**Results:**

The results suggest a pre-breeding program should be initiated directly from landraces. Initiating from testcrosses leads to a rapid reconstruction of the elite donor genome during further improvement of the pre-bridging germplasm. The analysis of accuracy of genomic predictions across the various design factors indicate the power of genomic selection for pre-breeding programs with large genetic diversity and constrained resources for data recording. The joint effect of design factors was summarized with decision trees with easy to follow guidelines to optimize pre-breeding efforts of SeeD and similar initiatives.

**Conclusions:**

Results of this study provide guidelines for SeeD and similar initiatives on how to initiate pre-breeding programs that aim to harness polygenic variation from landraces.

**Electronic supplementary material:**

The online version of this article (doi:10.1186/s12864-015-2345-z) contains supplementary material, which is available to authorized users.

## Background

This paper uses stochastic simulation to evaluate designs for initiating maize pre-breeding programs that harness polygenic variation from landrace populations for later incorporation into elite maize breeding populations. Today’s elite maize germplasms have lower genetic variance than progenitor populations [1–3], because they were sourced from a limited set of ancestral populations [4, 5] and to a smaller extent due to recent selection [6].

Such a reduced genetic variance limits the potential to breed for new market demands, new pathogens, and changing environments [7–11]. These breeding goals would be easier to address if the vast genetic variation of progenitor populations would be accessible to breeders in a form they could use in their breeding programs (e.g., see [12] and references within).

Extensive genetic variation is available in the diverse maize landrace populations around the globe [1–3] as a result of the open-pollinated reproductive system of maize and variation in its components [13], introgression from wild relatives [14], seed exchange between farmers, mutation, drift, and mild selection operating over a range of environments and time [13, 15, 16]. Some landraces are well adapted to extreme environments and it is likely they contain favorable alleles that could be used as a genetic resource to enrich the elite germplasms [17]. To use these resources breeders need to bridge the wide performance gap between landrace and elite germplasms, as landraces tend to have low performance, as well as high heterogeneity and negative genetic load. This process can be accelerated by using existing composite or recurrent selection populations, or even inbred lines derived from local landraces [12, 18].

**Fig. 1:**
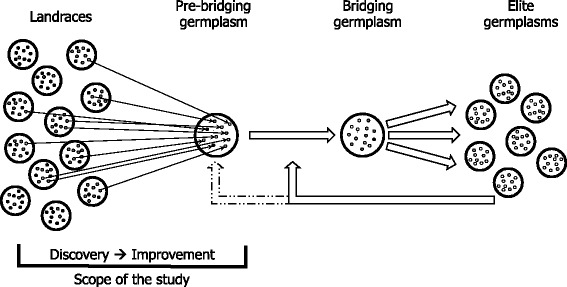
Scheme of a pre-breeding program in the Seeds of Discovery (SeeD) initiative

A recent initiative to characterize and use a part of the untapped variation in landraces is Seeds of Discovery (SeeD; http://seedsofdiscovery.org) funded mostly by the Mexican government through the Sustainable Modernization of Traditional Agriculture program (MasAgro; http://masagro.mx). SeeD aims to identify and enable use of favorable variation from landraces to develop bridging germplasm with 75 % or more elite and 25 % or less landrace genome (Fig. 1). This bridging germplasm is planned to provide donor lines carrying novel, landrace-derived genetic variation, to breed for high value characteristics such as nutritional quality, heat and drought tolerance, disease resistance, and tolerance to soil infertility. To this end the breeder’s core of 4,000+ maize landrace accessions from the germplasm bank housed at the International Maize and Wheat Improvement Center (CIMMYT) were genotyped with many markers and phenotyped for testcross performance (http://seedsofdiscovery.org). This resource provides one foundation for harnessing favorable variation from landraces.

Since the traits targeted by SeeD are predominantly polygenic e.g., [11, 19], data generated from this population can be approached both by genome-wide association mapping and by genomic selection. Genome-wide association mapping has and continues to be used in SeeD to highlight genomic regions with sizeable associations. Once such regions are identified and underlying alleles characterized, a limited number can be introgressed into elite germplasms following established forward breeding procedures [20–23].

Genomic selection offers an alternative paradigm where favorable genetic variation can be targeted across the whole genome and deleterious variation deselected, without focusing on few genomic regions, which is of particular value for traits of higher genetic complexity. In the context of a pre-breeding program genomic selection could be used to enrich the starting germplasm (from here onwards called as the pre-bridging germplasm) with favorable polygenic variation [24]. Such an enriched pre-bridging germplasm can then be used as a source for crossing with breeder proposed elite lines from the same heterotic group to create a bridging germplasm that contains 25 % or lower exotic genome to provide donor lines for introgression into elite germplasms (Fig. 1).

Despite the high potential value of genomic selection and forward breeding applications, there are many unknowns about how to initiate a pre-breeding program to optimize the outcome within the economic and logistical constraints. This is especially challenging in SeeD because of the vast genetic diversity addressed. An important opportunity for SeeD is the recent deployment of affordable high-throughput genomic tools for plant breeding [25, 26], which enable powerful analysis of the collected genomic and phenotypic data. This resource is expected to enable accurate genomic selection with short breeding cycles [27–29], which is essential for speeding up the improvement of pre-bridging germplasm. Genomic selection has been shown to speed up improvement within adapted × exotic crosses with more exotic germplasm introgressed per unit of time in comparison with phenotypic backcross selection [30, 31]. SeeD seeks to develop a bridging germplasm of donor lines for such crosses, but it is unclear how to use genomic selection paradigm to initiate such a pre-breeding program to harness sufficient amounts of polygenic variation from diverse populations, such as landraces.

This study aimed to evaluate the proposed designs to initiate a pre-breeding program within the SeeD initiative with emphasis on harnessing polygenic variation from landraces using genomic selection. The design factors included:

i) the approach to initiate a pre-breeding program from the selected landraces, doubled haploids of the selected landraces, or testcrosses of the elite hybrid and selected landraces,
ii) genetic parameters of landraces and phenotypes of interest, and
iii) logistical factors related to the size and management of a pre-breeding program.

## Methods

A pre-breeding program that would harness polygenic variation from landraces can be initiated in many possible ways. This research evaluated a preselected set of designs proposed within SeeD and quantified the effect of different design factors on the quality of the resulting pre-bridging germplasm (Fig. 1).

We quantified the effect of each design factor by a stochastic simulation, which involved generating: i) genomes of landraces and the elite hybrid (Additional file 1), ii) marker genotype data using the genotyping-by-sequencing (**GBS**) technology (Additional file 1), iii) trait genetic architecture (quantitative trait loci, **QTL**), true breeding values (**TBV**), and phenotypes (Additional file 1), iv) the discovery phase with a large landrace × elite training population and different approaches to initiate the pre-bridging germplasm, and v) the improvement phase with a synthetic population used to improve the pre-bridging germplasm. The simulation steps were performed by reusing the code base of AlphaDrop program [32], which lead to the development of AlphaSim program [33] available at http://www.alphagenes.roslin.ed.ac.uk/alphasuite/alphasim. The simulation outputs were analyzed using R [34].

### Discovery phase

The aim of the discovery phase was to discover the best landraces and the best seeds within these landraces to initiate the pre-bridging germplasm. Ranking of landraces and seeds within a landrace was based on their estimated breeding value (**EBV**) for testcross performance inferred from the collected genomic and phenotypic information. This phase was conducted in three stages that involved i) generating landrace × elite training population, ii) selecting the best landraces, and iii) selecting the best seeds within the selected landraces. The first stage involved generating the training population by first growing one, three, or five sampled seeds from each of the 3,000 landraces, and then crossing them with the elite hybrid to get a population of 3,000, 9,000, or 15,000 testcrosses, respectively. The obtained testcross phenotypes were regressed on the corresponding landrace seed marker genotypes using ridge regression to train a genomic prediction equation [27, 35, 36]. The prediction equation was used to get an EBV for each planted seed per landrace and a mean EBV for each landrace, as well as EBV for non-phenotyped but genotyped seeds when required. The second stage involved selecting the best 40 or 80 landraces according to their mean EBV. The third stage involved genotyping 10, 20, or 40 random seeds from the selected landraces (in total between 40 × 10 = 400 and 80 × 40 = 3,200 seeds) and selecting the best 10 according to their EBV, with a restriction that only one seed per landrace was selected.

The third stage, selecting the best seeds, followed one of the three different approaches of initiating the pre-bridging germplasm. The Landrace approach was based on initiating from the selected seeds of the selected landraces (Additional file 2: Figure S1). The LandraceDH approach was like the Landrace approach, but extended to initiate from the best doubled haploids of the selected seeds to get inbred starting individuals (Additional file 2: Figure S2). To start with the landrace genome segments introgressed into the elite background the LandraceElite approach was based on initiating from the selected testcross seeds of the selected landrace × elite crosses (Additional file 2: Figure S3).

### Improvement phase

The aim of the improvement phase was to further improve the initiated pre-bridging germplasm from the discovery phase. This phase involved four stages with a breeding cycle within each. The first breeding cycle was started by planting the selected seeds from the discovery phase and crossing the resulting plants at random to initiate a synthetic population (Additional file 2: Figure S4). Ten seeds from each of the ten grown plants were sampled for genotyping (100 altogether) and their EBV were calculated using the available prediction equation. The best ten seeds were selected based on their EBV to start a new breeding cycle. A random sample of 0, 20, 40, or 60 of the remaining seeds were grown and crossed to the elite hybrid to obtain their testcross performance data for retraining the genomic prediction equation. The new prediction equation was obtained by regressing the obtained testcross phenotypes on the corresponding synthetic seed genotypes collected within a cycle using ridge regression as in the discovery phase. The discovery data were excluded from retraining. Since two generations were needed to obtain testcross phenotypes, the updated equation from a cycle could only be used in the next cycle.

### Analysis

This study simulated and analyzed the effect of the following design factors:

i. the approach to initiate the pre-bridging germplasm (Landrace, LandraceDH, or LandraceElite),
ii. genetic parameters - the diversity of the founding population (effective population size (*N*_*e*_) of 1,000 or 100,000), the diversity within landrace accessions (inbreeding coefficient (*F*) of 0.3 or 0.9), and the heritability of the selection trait (*h*^2^ =0.25 or *h*^2^ =0.50) and
iii. logistical factors - the genotyping platform (high coverage GBS with 10,000 markers (GBS10*x*@10K) or low-coverage GBS with 100,000 markers (GBS1*x*@100K)), number of phenotyped seeds per landrace in the discovery phase (1, 3, or 5), the number of selected landrace (40 or 80), the number of tested seeds per selected landrace (10, 20, or 40), and the number of phenotyped synthetic seeds for retraining in the improvement phase (0, 20, 40, or 60).

Altogether 3,456 different scenarios were replicated ten times. The simulation outputs are available in the R data file format [34] in the Additional file 3.

For each scenario genetic merit, accuracy of genomic prediction, kinship with the elite hybrid, and heterozygosity of germplasm were collected in different stages of the discovery and improvement phases. Here germplasm represents a collection of individuals, i.e., landraces, selected landraces, or the whole pre-bridging germplasm. Genetic merit was expressed relative to the mean genetic merit of landraces as well as to the elite hybrid individual so that the mean genetic merit of landraces was zero and the genetic merit of the elite hybrid was one. This was achieved by first computing the mean TBV of landraces, which was subtracted from the mean TBV of a germplasm and from the TBV of the elite hybrid. Then, the subtracted mean TBV of a germplasm was divided by the subtracted TBV of elite hybrid. Accuracy was measured as the correlation between EBV and TBV. Kinship with the elite hybrid was measured as the proportion of 1 cM identical genome segments between a germplasm and the elite hybrid to determine uniqueness of the germplasm in comparison with the elite hybrid. Heterozygosity was measured as mean heterozygosity over segregating sites to determine the genetic diversity of the germplasm, which indicates its potential for further improvement.

It was hypothesized that the approach of initiating the pre-bridging germplasm would substantially affect the genetic merit and uniqueness of such a germplasm and successful initiation of the pre-breeding program. Therefore, the analysis focused on the approach to initiate the pre-bridging germplasm as a main effect and on the interaction with the genetic parameters of landraces and phenotypes. These parameters described a range of possible landraces from which a pre-breeding program might aim to harness polygenic variation. Once the effect of the three approaches was determined, the most promising approach was analyzed in detail. The detailed analysis assessed the effect of different design factors on genetic merit in the final stage of the discovery and improvement phases for a subset of scenarios that are relevant for SeeD, i.e., a very diverse founding population (*N*_*e*_ = 100,000), and high genetic diversity within landrace accessions (*F* = 0.3). The simulation output and the accompanying easy to use R code [34] in the Additional file 3 can be used to explore other subsets of scenarios.

The effect of each design factor was first evaluated via a linear model [34, 37]. Then a decision support system was developed using the regression tree analysis, which builds a decision tree by recursive partitioning the modeled variable according to the most influential design factor on each iteration, taking into account both main and interaction effects [38, 39]. Both the linear model and regression tree analyses were based on modeling the genetic merit of pre-bridging germplasm in different scenarios as a function of heritability, genotyping platform, the number of phenotyped seeds per landrace, the number of selected landraces, the number of tested seeds per selected landrace, and the number of phenotyped synthetic seeds. Differences between the levels of design factors were declared significant at the *p* < 0.01 level in both the linear model and regression tree analysis.

## Results

### Comparison of approaches of initiating the pre-bridging germplasm

**Fig. 2:**
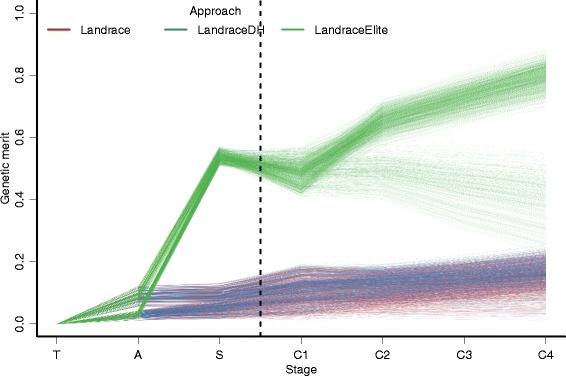
Genetic merit in different stages by approach. Discovery phase (training – T, the selected accessions – A, the selected seeds from the selected accessions – S). Improvement phase (the selected seeds in each of the four cycles – C1-C4). One line represents a mean over ten replicates

The approach to initiate the pre-bridging germplasm significantly affected the genetic merit of the resulting germplasm. The LandraceElite approach gave a pre-bridging germplasm with the highest mean genetic merit of 0.54 at the start of improvement phase (Fig. 2, Additional file 2: Table S1), which was mostly due to advancing half of the elite hybrid genome (the genetic merit of the elite hybrid individual was 1.00 by definition) and only 0.04 due to advancing the favorable genetic variation from landraces. In comparison, the Landrace and LandraceDH approaches both gave a pre-bridging germplasm with a mean genetic merit of 0.07 at the start of improvement phase. By the end of improvement phase, the genetic merit was 0.70 with the LandraceElite approach, 0.16 with the LandraceDH approach, and 0.15 with the Landrace approach.

Two sets of LandraceElite scenarios had distinct trajectories of genetic merit throughout the improvement phase reflecting retraining of the prediction equation. The first set of scenarios, where re-training was omitted, showed a slow but continuous decrease in the mean genetic merit to 0.40, in contrast the scenarios where re-training was performed showed a steady increase in the mean genetic merit to 0.80.

The influence of both diversity of founding populations and heritability did not show strong interactions (Additional file 2: Fig S5). Genetic merit was higher when the diversity of the founding population was low (*N*_*e*_ = 1,000), when the diversity within landrace accessions was high (*F* = 0.3) or when the heritability was high (*h*^*2*^ = 0.50).

**Fig. 3:**
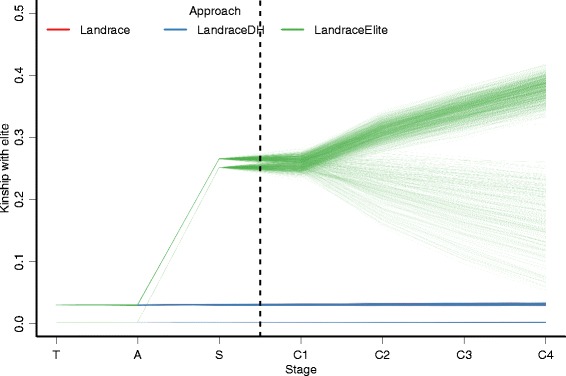
Kinship with the elite hybrid in different stages by approach. Discovery phase (training – T, the selected accessions – A, the selected seeds from the selected accessions – S). Improvement phase (the selected seeds in each of the four cycles – C1-C4). One line represents a mean over ten replicates

The kinship with the elite hybrid was used to describe the distinctiveness of pre-bridging germplasm. For both the Landrace and LandraceDH approaches the mean kinship was only 0.02 in the training population and stayed at this level through all stages (Fig. 3, Additional file 2: Table S2). The LandraceElite approach increased mean kinship with the elite hybrid to 0.26 after selecting among the landrace × elite seeds and to 0.32 by the end of improvement phase. The kinship value after selection was close to the expected value of 0.25; the probability of choosing an allele identical by descent from the elite hybrid and its progeny is 25 %. These results indicate that the LandraceElite approach was increasing the frequency of the elite haplotypes throughout the improvement phase, i.e., it was reconstructing the elite genome. This was not the case for a set of LandraceElite scenarios with omitted retraining of the prediction equation; there the mean kinship with the elite hybrid decreased to 0.17 by the end of the improvement phase. When the prediction equation was retrained, the mean kinship with the elite hybrid increased to 0.38 by the end of the improvement phase. Apart from larger values when the diversity of the founding population was low, no strong interactions were found between the approach and the genetic parameters on kinship with the elite hybrid (Additional file 2: Figure S6). The larger kinship values with low diversity of the founding population can be attributed to a higher chance of seeing the same haplotype in both the elite hybrid and landrace accessions.

**Fig. 4:**
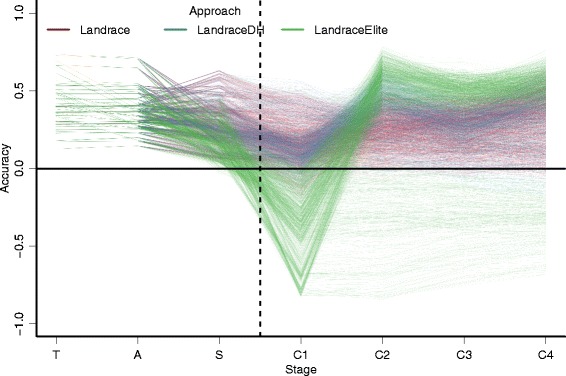
Accuracy of genomic evaluation/prediction in different stages by approach. Discovery phase (training – T, the selected accessions – A, the selected seeds from the selected accessions – S). Improvement phase (the selected seeds in each of the four cycles – C1-C4). One line represents a mean over ten replicates

The mean accuracy of genomic prediction was 0.42 in the training stage and 0.37 when selecting the best landraces (Fig. 4, Additional file 2: Table S3). For the training stage, accuracy was defined as a correlation between the EBV and TBV of phenotyped landrace (training) seeds, while for selecting the best landraces accuracy was defined as a correlation between the mean EBV of training landrace seeds and the mean TBV of the landrace accession seeds. The approach to initiate pre-bridging germplasm significantly affected the accuracy of selecting the best seeds from the selected landraces. The mean accuracy was 0.28 for both the Landrace and LandraceDH approaches and 0.18 for the LandraceElite approach. The approaches differ due to the elite hybrid haplotypes not being included in the training of the prediction equation, i.e., only landrace genotypes were included in the training population.

Even larger differences in the mean accuracy between the approaches were observed in the first stage of the improvement phase; the mean accuracy was 0.16 for the Landrace approach, 0.14 for the LandraceDH approach, and −0.33 for the LandraceElite approach. Differences between the approaches reduced after the first round of retraining the prediction equation. The three approaches had comparable accuracies in the final stage of the improvement phase (0.32 with the Landrace approach and 0.33 with both the LandraceDH and LandraceElite approach).

Two sets of LandraceElite scenarios had distinct trajectories of accuracies throughout the improvement phase. The first set of scenarios showed no improvement in accuracy after the initial drop and stayed at the same value of −0.30 throughout the improvement phase because the retraining of prediction equation was omitted for these scenarios. The second set of scenarios showed a fast increase in mean accuracy to 0.54, which was due to retraining the prediction equation. The interaction between the approach to initiate pre-bridging germplasm, the diversity of the founding population, the diversity within landrace accessions, and the heritability affected accuracy of genomic prediction (Additional file 2: Figure S7). The pattern of change in accuracies was the same for the different combinations of genetic parameters (Fig. 4), but was more pronounced when the diversity of the founding population was high and the diversity within landrace accessions and the heritability were low, particularly for the LandraceElite approach. These differences were due to the effect of genetic parameters on the accuracy of genomic prediction and the elite hybrid haplotypes excluded from training. For example, with the LandraceElite approach the accuracy was −0.02 in the first stage of improvement phase when the diversity of the founding population was low (*N*_*e*_ = 1,000), the diversity within landrace accessions was low (*F* = 0.3), and the heritability was high (*h*^*2*^ = 0.50), while it was −0.54 when the diversity of the founding population was high (*N*_*e*_ = 100,000), the diversity within landrace accessions was high (*F* = 0.9), and the heritability was low (*h*^*2*^ = 0.25) (Additional file 2: Figure S7).

**Fig. 5:**
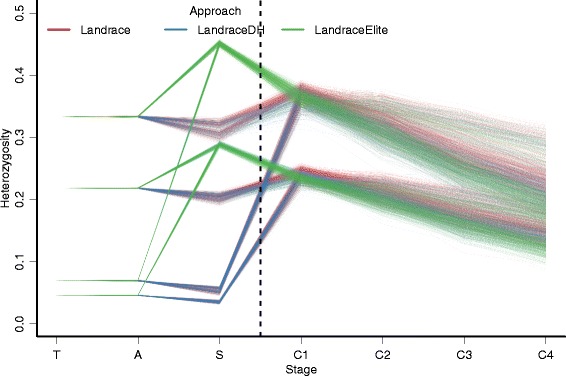
Heterozygosity in different stages by approach. Discovery phase (training – T, the selected accessions – A, the selected seeds from the selected accessions – S). Improvement phase (the selected seeds in each of the four cycles – C1-C4). One line represents a mean over ten replicates

The heterozygosity of germplasm was also influenced by the approach to initiate pre-bridging germplasm (Fig. 5, Additional file 2: Table S4). Landrace accessions had a mean heterozygosity of 0.17 and selecting the ones with the highest genetic merit did not change this measure of genetic diversity. Due to selection the heterozygosity decreased slightly (to 0.15) in the selected seeds for both the Landrace and LandraceDH approach. In contrast, the introgression of the landrace alleles into the elite background caused the heterozygosity to increase substantially (to 0.37) in the selected seeds for the LandraceElite approach. The heterozygosity increased in the first stage of the improvement phase with the Landrace and LandraceDH approaches due to the mixing of genomes of the different landraces and decreased thereafter due to selection. Heterozygosity decreased with the LandraceElite approach throughout the improvement phase because of selection. In the final stage of the improvement phase the heterozygosity was highest with the Landrace approach (0.19), followed by the LandraceDH approach (0.18), and then the LandraceElite approach (0.17).

Some LandraceElite scenarios had comparable heterozygosity to the Landrace and LandraceDH approaches, due to inaccurate selection caused by the lack of retraining the prediction equation. The interaction between the approach to initiate pre-bridging germplasm, the diversity of the founding population, and diversity within landrace accessions affected heterozygosity (Additional file 2: Figure S8). The mean heterozygosity of accessions was expected to be low when diversity within accessions was low (*F* = 0.9). However, the mixing of divergent genomes caused the mean heterozygosity to increase substantially in the selected landrace × elite seeds or in the first stage of synthetic seeds with the Landrace and LandraceDH approaches.

The comparison of the different approaches to initiate pre-bridging germplasm shows significant differences between the LandraceElite and either the Landrace or LandraceDH approaches and only a small difference between the Landrace and LandraceDH approaches. The LandraceElite approach differs because the pre-bridging germplasm is initiated with the selected landrace × elite individuals that contain 50 % elite genome. Further improvement of this germplasm leads to the rapid reconstruction of the elite genome. This reconstruction limits the enrichment of pre-bridging germplasm with valuable landrace alleles and is thus unaligned with the goals of SeeD. Further analyses were focused on the effect of design factors using the Landrace approach only.

### Marginal effect of design factors for the Landrace approach for SeeD

The tested design factors affected the genetic merit of pre-bridging germplasm at the end of the discovery and improvement phases (Table 1). Higher heritability (0.25 vs. 0.50) gave pre-bridging germplasm with higher genetic merit (0.033 vs. 0.045 in the discovery phase and 0.098 vs. 0.130 in the improvement phase). Differences of similar size were observed when using larger numbers of GBS markers with low coverage (GBS1*x*@100K) in comparison with smaller numbers of GBS markers with higher coverage (GBS10*x*@10K). This suggests that initiation and improvement of pre-bridging germplasms should be done with more GBS markers even if they have lower accuracy of called genotypes.

**Table 1:** Genetic merit (mean and 95 % quantiles over scenarios and replicates^*^) in the final stage of discovery and improvement phase with the Landrace approach, high diversity of the founding population (*N*_*e*_ = 100,000), and high diversity within landrace accessions (*F* = 0.3) by the levels of different factors

Level	Discovery phase	Improvement phase
Heritability		
0.25	0.033 (0.012, 0.056)^a^	0.098 (−0.005, 0.200)^a^
0.50	0.045 (0.020, 0.072)^b^	0.130 ( 0.014, 0.232)^b^
Genotyping platform		
GBS10x@10K	0.029 (0.011, 0.049)^a^	0.098 (−0.014, 0.210)^a^
GBS1x@100K	0.050 (0.030, 0.072)^b^	0.130 ( 0.031, 0.232)^b^
Number of phenotyped seeds per landrace		
1	0.035 (0.013, 0.061)^a^	0.109 (−0.008, 0.219)^a^
3	0.040 (0.014, 0.069)^b^	0.116 ( 0.008, 0.223)^b^
5	0.042 (0.017, 0.071)^c^	0.117 ( 0.009, 0.224)^b^
Number of selected landraces		
40	0.042 (0.016, 0.072)^a^	0.115 ( 0.003, 0.220)^a^
80	0.036 (0.013, 0.063)^b^	0.113 ( 0.001, 0.226)^a^
Number of tested seeds per selected landrace		
10	0.038 (0.014, 0.066)^a^	0.113 (−0.004, 0.228)^a^
20	0.040 (0.014, 0.068)^b^	0.114 ( 0.000, 0.218)^a^
40	0.041 (0.015, 0.070)^c^	0.114 ( 0.009, 0.222)^a^
Number of phenotyped synthetic seeds		
0	/	0.069 (−0.020, 0.166)^a^
20	/	0.116 ( 0.016, 0.213)^b^
40	/	0.131 ( 0.036, 0.229)^c^
60	/	0.139 ( 0.038, 0.244)^d^

^*^Means with different letter within a column and a factor are different at *p* < 0.01

The number of phenotyped seeds per landrace also affected the genetic merit of pre-bridging germplasm. The mean genetic merit with one, three, and five seeds per landrace were 0.035, 0.042, and 0.040, respectively by the end of the discovery phase and 0.109, 0.117, and 0.116, respectively by the end of the improvement phase. The number of selected landraces and the number of tested seeds per selected landrace affected the end of the discovery phase, but this effect was diluted by the end of the improvement phase. Selecting more landraces initiated a pre-bridging germplasm with lower genetic merit (0.042 and 0.036 with 40 and 80 selected landraces, respectively), while testing more seeds per landrace increased the intensity of selection and initiated a pre-bridging germplasm with higher genetic merit (0.038, 0.040, and 0.041 with 10, 20, and 40 tested seeds per landrace, respectively). The number of phenotyped synthetic seeds in the improvement phase affected the genetic merit of resulting germplasm. When the synthetic population was unphenotyped and the retraining was omitted the resulting germplasm had low mean genetic merit (0.069). Phenotyping at least 20 synthetic seeds and retraining the prediction equation increased the genetic merit of the final pre-bridging germplasm to 0.116. Increasing the number of phenotyped synthetic seeds to 40 or 60 increased the genetic merit of the final pre-bridging germplasm to 0.131 or 0.139, respectively.

### Decision support system for the Landrace approach for SeeD

**Fig. 6:**
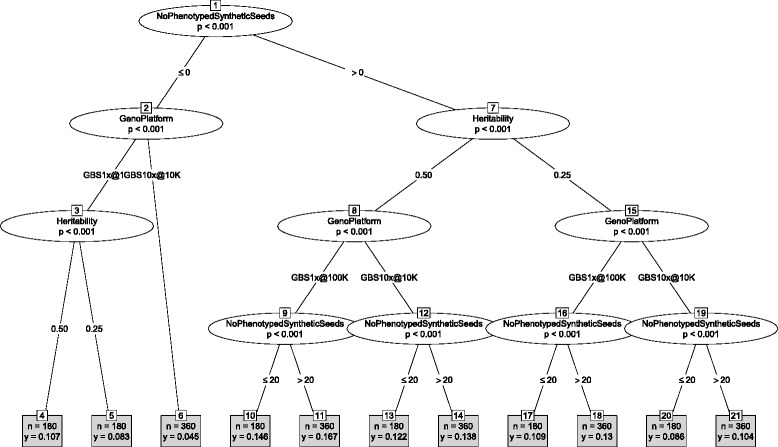
Decision tree for genetic merit in the final stage of discovery phase. With the Landrace approach, high diversity of the founding population (Ne = 100,000), and high diversity within accessions (F = 0.3)

Genetic merit of the pre-bridging germplasm was affected by many interactions between the design factors. The summary of the interaction effects on the genetic merit of selected seeds from the selected landraces at the end of the discovery phase is shown with the decision tree in Fig. 6. In that stage the design factor with the largest effect was the genotyping platform, followed by the trait heritability. The number of selected landraces, the number of tested landrace seeds, and the number of phenotyped landrace seeds interacted with each other and with the genotyping platform and the trait heritability in different ways. Despite the interactions a decision tree provides easy to follow guidelines. For example, the strategy to identify the best landrace seeds for a trait with the heritability of 0.25 was to use 100,000 GBS markers with 1*x* coverage, test more than 10 seeds from 40 selected landraces. The observed differences in genetic merit of the selected seeds are the result of differences in selection intensity and accuracy of genomic prediction when selecting landraces and seeds within these landraces (Additional file 2: Figure S9-S10).

**Fig. 7:**
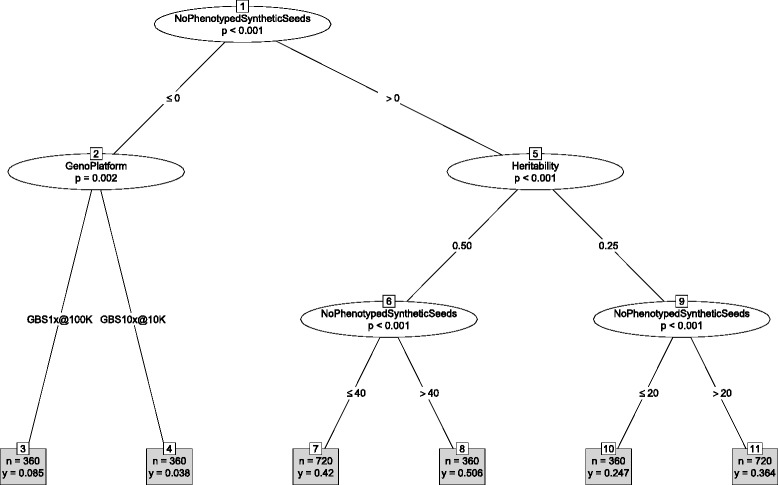
Decision tree for genetic merit in the final stage of improvement phase. With the landrace approach, high diversity of the founding population (Ne = 100,000), and high diversity within accessions (F = 0.3)

While many design factors affected the genetic merit of pre-bridging germplasm at the end of the discovery phase (Fig. 6), few factors had a lasting effect until the end of the improvement phase (Fig. 7). This suggests that some of the main effects and their interactions were canceled out during the discovery and improvement phases. The design factor with the largest effect on the genetic merit of final germplasm was the number of phenotyped synthetic seeds, followed by the genotyping platform and the trait heritability. The same design factors determined the accuracy of genomic prediction in the improvement phase (Additional file 2: Figure S11). The strategy to develop germplasm with the highest genetic merit for a trait with heritability of 0.25 or 0.50 was to phenotype more than 20 synthetic seeds and use 100,000 GBS markers with 1*x* coverage (Fig. 7). Such a strategy would lead to a germplasm with a mean genetic merit of 0.130 and 0.167 for a trait with heritability of 0.25 and 0.50, respectively; reducing the number of phenotyped synthetic seeds to 20 would lower the mean genetic merit to 0.109 and 0.146, respectively. Omitting phenotyping and retraining in the improvement phase would decrease the mean genetic merit to 0.083 and 0.107.

## Discussion

Genetic variation for traits of interest is the fundamental building block of conventional crop improvement strategies. The limited genetic diversity in elite maize germplasms raises concerns about the genetic potential to address existing and new breeding goals. Several initiatives, such as SeeD, are addressing this problem by searching for favorable variation in diverse landraces. The SeeD initiative aims to identify and extract favorable variation from gene bank accessions in Mexico and develop a semi-elite bridging germplasm, which breeders can use for increasing diversity of their breeding programs. This study evaluated the proposed genomic selection designs to initiate a precursor of such a bridging germplasm, the pre-bridging germplasm, to be further improved and developed into the final bridging germplasm (Fig. 1). The study evaluated the effect of three categories of design factors on the genetic merit of resulting pre-bridging germplasm. The first category consisted of three approaches to initiate pre-bridging germplasm from either the selected landraces, doubled haploids derived of the selected landraces, or testcrosses of the selected landraces and elite hybrid. The second category represented different genetic parameters of landraces and phenotypes. The third category represented logistical factors about the size and management of the pre-breeding program.

### Comparison of approaches of initiating the pre-bridging germplasm

The results show that the approach to initiate the pre-breeding germplasm has a significant effect on the outcome. Highest genetic merit was achieved by initiation with testcrosses. However, this high merit was achieved by reconstructing the elite genome and not by utilizing the favorable variation in landraces and as such was contrary to the aims of pre-breeding. A potential way to avoid reconstructing the elite genome would be to randomly mate the initial landrace × elite individuals for several generations to recombine the elite and landrace segments, breaking the linkage between the favorable and unfavorable alleles in the landrace segments e.g., [12, 30]. However, such mixing would take several generations. In addition, it will only partially solve the main problem, which is that the high frequency of favorable alleles from the elite genome mask the effect of those in landrace genomes [12]. While this is a minor issue when introgressing single alleles with major effects, it becomes a problem when the number of alleles increases, the effect of alleles decreases, and the gap between the landrace and elite germplasms widens. Thus, the common approach of mining for favorable alleles and introgressing them into the elite germplasms [40, 41] is unfeasible for polygenic traits for at least two reasons. First, the power to identify loci underpinning polygenic traits is low with the currently available resources. Huge sets of phenotyped and densely genotyped populations will be needed to mine for such loci [42, 43]. Until data sets of such size are available, selection of high merit individuals will be based on associations between variation in large genome segments and variation in phenotypes [29, 44–47]. Second, even if such loci and their alleles are identified, the main problem of bridging the wide gap between landrace and elite germplasms remains unsolved.

The more promising approach of pre-breeding for polygenic traits is to avoid too early introgression into elite backgrounds by initiating a pre-bridging germplasm from the most promising landraces as proposed within SeeD with the Landrace or LandraceDH approaches. The pre-bridging germplasm should then be improved independently until the frequency of favorable alleles is increased. This approach is in line with existing experiences of introgressing exotic material into the adapted germplasms, i.e., introgression is generally more successful with an advanced exotic material than with the diverse landraces [12, 18]. The Latin America Maize Project (LAMP) [48] and the Germplasm Enhancement of Maize (GEM) program [7, 49] provide an example of such an approach. In LAMP many landraces across Latin America were tested and the most promising ones were used in the GEM program with the aim of releasing promising advanced material for further breeding [50, 51]. The scope of our study aligns partially with the first phase of phenotypic screening for the most promising landraces in LAMP, but we used genomic selection for screening and further improvement of the pre-bridging germplasm.

Improvement of the pre-bridging germplasm initiated from landraces can be slow and costly. An opportunity for SeeD is the recent emergence of affordable high-throughput genotyping for plant breeding [25, 26], which complements phenotyping activities in SeeD. These resources provide a foundation for a genomic pre-breeding program with genomic selection and much shorter breeding cycles than with standard phenotype based programs [28, 29, 52]. In this study, the mean response to selection achieved with was equal to 2.52 genetic standard deviations (GSD) in 6 seasons with the Landrace approach, and 2.68 GSD in 9 seasons with the LandraceDH approach considering the generation of double-haploids (assuming one season of phenotyping in each case). In comparison, the LandraceElite approach was much better with 16.78 GSD above the mean of landrace accessions. However, given the large diversity of the founding population and among the landrace accessions and quite modest size of the training population for such a diverse setting, the responses achieved are considerable. The response per unit of time was 0.42 GSD with the Landrace approach and 0.30 GSD with the LandraceDH approach. Beside the smaller response to selection per unit of time in this study, the LandraceDH approach would have an additional drawback due to the high genetic load of recessive deleterious alleles in landraces, which would limit the success of generating doubled haploids. This suggests that at the early stage of pre-breeding the use of doubled haploid technology provides little benefit for breeding for higher additive genetic merit. However, doubled haploid technology can be effective in later stages for rapid development of the more advanced lines and for purging the (pre-)bridging germplasm of deleterious alleles before its further improvement or introgression into the elite backgrounds [53].

### Accuracy of genomic predictions

The results show the power of genomic selection in diverse populations, e.g., landraces, and as such they expand the scope of previous genomic selection studies within a cross of elite [28] or exotic × adapted [30] material, and across crosses e.g., [29, 46, 54, 55]. Indeed, this study covered a wide spectrum of settings that are of interest for evaluating the accuracy of genomic prediction in a diverse population from the discovery phase to the early generation synthetic population in the improvement phase. Seven stages across both phases had different settings that affected accuracy: training, selecting the best landraces, selecting the best seeds from the selected landraces in the discovery phase, and selecting the best synthetic seeds in the four stages of the improvement phase.

The first stage, training, estimated parameters of a genomic prediction equation in a diverse landrace × elite training population by regressing the landrace × elite phenotypes on the marker genotypes of planted landrace seeds. Mean accuracy of evaluating the genetic merit of these seeds was 0.42, which is high for such a diverse setting, i.e., the effective population size of the founding population was either 1,000 or 100,000, but this is expected for individuals from within a training population.

The second stage, selecting the best landraces, used the prediction equation from the first stage. Mean accuracy of predicting the mean genetic merit of landrace accessions was 0.37, which is also high for such a diverse setting and when the prediction is being done outside of the training population. High accuracy at this stage was achieved because group means vary less and more information was available to estimate them, making it is easier to predict the mean value of a group of individuals than the value of a particular individual within a group. The level of accuracy is in line with the accuracy in a set of wheat landraces [56].

The third stage, selecting the best seeds from the selected landraces with the Landrace and LandraceDH approach or from the selected crosses with the LandraceElite approach, again used the prediction equation from the first stage. Mean accuracy of this prediction was 0.28 with the Landrace and LandraceDH approaches and 0.18 with the LandraceElite approach. These results confirm that predicting an individual’s genetic merit is indeed harder, especially if predicting the genetic merit of a preselected set of individuals, i.e., seeds from the selected landraces. These accuracies are higher than the theory of genomic prediction suggests it should be for a setting with so large genetic diversity [57, 58]. This deviation from the theory was due to the close relationships between the training and prediction individuals [29, 44, 45, 47], which the theory does not account for. However, the results are in agreement with the results of [59, 60], where high accuracies were also found with diverse populations of training individuals and close related prediction individuals. Accuracy in our study was lower with the LandraceElite approach than with the Landrace approach, because the landrace × elite seeds were less related to the training population (the elite genome was excluded from training) than the landrace seeds.

The fourth stage, selecting the best first-generation synthetic seeds, used the prediction equation from the first stage. At this stage, the difference in accuracy between the approaches was large. Mean accuracy at this stage was 0.16 with the Landrace approach and 0.14 with the LandraceDH approach. This large drop in comparison with the third stage was due to the further increase in distance from the training population. The small, but significant, difference between the Landrace and LandraceDH approaches was caused by the doubled haploid step, which changed the genetic variance of prediction population relative to the training population. Mean accuracy with the LandraceElite approach was, however, −0.33, i.e., individuals with high genetic merit got, on average, a low prediction. This stark drop in accuracy arose because the mixing of elite hybrid and landrace genomes “broke” associations between the markers and QTL estimated in the landrace training population. Accuracy was negative because the variation in landrace training population involved favorable and unfavorable QTL alleles in combinations with the marker genotypes, while the elite genome was enriched for favorable QTL alleles in different combinations with the marker genotypes. In other words, genetic variation in the landrace training population was on average in repulsion with the variation in the elite background. Consequently, the response to selection was negative in the first stage of the improvement phase with the LandraceElite approach and remained negative throughout the improvement phase if retraining was omitted.

The fifth stage, selecting the best second-generation synthetic seeds, used the retrained prediction equation from the first-generation synthetic seeds. Mean accuracy at this stage was 0.26 with the Landrace approach, 0.33 with the LandraceDH approach, and 0.34 with the LandraceElite approach. The Landrace approach had the lowest accuracy as a result of the higher genetic diversity. The LandraceDH had lower diversity due to selection of doubled haploids, while the LandraceElite had lower diversity due to the half of genome originating from the elite hybrid parent. These accuracies are somewhat comparable in value to those of selecting the best landraces (the second stage) and selecting the best seeds from the selected landraces (the third stage), but the setting was different. In the discovery phase the genetic diversity was high and the training population was large (3,000, 9,000, or 15,000 individuals), while in the improvement phase the genetic diversity was reduced due to selection and the training population was smaller (only 0, 20, 40, or 60 individuals).

In the sixth stage, selecting the best third-generation seeds, and in the seventh stage, selecting the best fourth-generation seeds, the accuracies with the three approaches were similar.

### Effect of design factors

Accuracy and the achieved genetic merit were affected by many pre-breeding program design factors evaluated in this study. Beside the approach to initiate pre-bridging germplasm and the stage of the pre-breeding program, there were also genetic and logistical factors. The genetic parameters affected accuracy in line with the theory [57, 58] – accuracies were higher with the higher heritability and with the lower effective population size. The logistical factors had variable effects throughout the simulation. The genotyping platform was based on GBS [25] with 10*x* coverage at 10,000 markers (GBS10*x*@10K) or with 1*x* coverage at 100,000 markers (GBS1*x*@100K) and the results showed that more markers with lower coverage gave higher accuracies in every stage of a pre-breeding program. This is an expected result because a large number of markers is required to capture the large genetic diversity in landraces [61]. The accuracy of called genotypes in GBS data depends on the coverage with higher coverage giving higher accuracy but also higher relative costs [25, 62, 63]. However, the results of this simulation show that low coverage (1*x*) genotype data can deliver accurate genomic predictions at low cost, as observed in [64, 65].

The effect of number of phenotyped seeds per landrace accession in the training set was in line with expectations with larger training populations leading to higher accuracies, but with large diminishing returns. Taking into account the costs it seems that of phenotyping only one seedling per accession might be enough as long as the total size of the training population enables accurate genomic predictions. It is unlikely that of phenotyping just one seedling per accession will represent a sizeable proportion of variation within each landrace, but this might provide some overlap between accessions to cover a fair share of total variation. The simulation suggests that a sizeable proportion of total variation was captured, as genomic predictions were accurate even for a setting with high diversity. However, these results might be too optimistic as the simulation of landraces was likely too simplistic [66].

The number of selected best landraces and the number of tested seeds per selected landrace increased response to selection with more intense selection as expected. However, this effect did not extend to the last stage of the simulation. This can be explained by the fact that increased intensity of selection reduced diversity, which in turn reduced the long-term response to selection. This result indicates that pre-breeding programs might benefit by using optimum contribution selection, which balances the short-term response to selection and the loss of genetic diversity to maximize the long-term response to selection [58, 67–69]. While large responses in short time scales can be expected for traits affected by few loci with large effects, such large responses are impossible for polygenic traits. It might be tempting to increase the intensity of selection to maximize the response to selection when developing pre-bridging germplasm for polygenic traits. This may lead to a rapid loss of diversity and creation of selection bottlenecks e.g., [4–6], which would need to be addressed with a higher number of separate and diverse parallel pre-breeding populations and higher costs. Finally, the results show that retraining the prediction equation is needed for the improvement of pre-bridging germplasm, as the accuracy of genomic prediction decreases rapidly with decreasing relationship between training and prediction individuals [29, 44, 45, 47].

Several design factors and their interactions affected the quality of resulting pre-bridging germplasm. To create easy to follow guidelines in such a setting we used decision trees. A decision tree is estimated from the variation of a modeled variable (for example the genetic merit of germplasm) in relation to other variables (for example design factors) that might influence the modeled variable on its own or through a complicated interaction [38, 39]. The estimated decision trees form an easy to follow decision support system for SeeD and similar initiatives in maize and other crops e.g., [70]. For example, a manager can use the decision tree to balance costs against expected gains to maximize return on investment.

The developed decision trees suggest that only a small number of the design factors evaluated in this study significantly affect the resulting pre-bridging germplasm, in particular the amount of collected phenotypes in the improvement phase, heritability, and genotyping platform. This study evaluated only a small subset of possible designs to initiate a pre-breeding program with a preselected subset of scenarios proposed within SeeD. Many more design factors than could have been evaluated in this study are likely to influence pre-breeding programs.

## Conclusions

The success of a pre-breeding program that aims to harness favorable polygenic variation from landraces will be largely affected by the approaches used to initiate the program. In particular interesting landrace haplotypes harboring polygenic variation should not be introgressed into elite backgrounds too early because the subsequent improvement will favor the elite haplotypes and limit the distinctness of resulting germplasm. Early introgression is feasible for loci with large effects, but not for polygenic loci. For the latter the focus should be put on genomic selection to increase frequencies of favorable alleles and with this bridge the gap between the landrace and elite germplasms. Pre-breeding programs are affected by many factors, whose effects can be summarized with decision trees to provide easy to follow decision support systems.

### Availability of supporting data

The simulation outputs supporting the results of this article are available in the Additional file 3.

## Additional files

Additional file 1:
**Detailed description of generating i) genomes of landraces and the elite hybrid, ii) marker genotype data, and iii) trait genetic architecture, quantitative loci, true breeding values, and phenotypes.** (PDF 424 kb) Additional file 2:
**Fig. {S1, S2, S3}.** Scheme of the discovery phase with the {Landrace,LandraceDH,LandraceElite} approach; **Fig. S4.** Scheme of the improvement phase; **Fig. {S5, S6, S7, S8}.** {Genetic merit, Kinship with the elite hybrid, Accuracy of genomic evaluation/prediction, Heterozygosity} in different stages by approach, genetic diversity of the founding population, genetic diversity within accessions, and heritability; **Fig. {S9, S10, S11}.** Decision tree for accuracy of {selecting accessions in the discovery phase, selecting seeds from the selected accessions in the discovery phase, in the final stage of improvement phase}; **Table {S1, S2, S3, S4}.** {Genetic merit, Kinship with the elite hybrid, Accuracy of genomic evaluation/prediction, Heterozygosity} in different stages by approach, genetic diversity of the founding population, genetic diversity within accessions, and heritability. (PDF 4858 kb) Additional file 3:
**Simulation outputs and accompanying R code for its analysis.** Analysis and decision support system for comparing simulation outputs of different designs of initiating a pre-breeding program from maize landrace populations. The ZIP file contains three files: SimulationOutput.RData – Simulation outputs in the R data file format, SimulationFunctions.R – R functions used for analysis, SimulationAnalysis.R – R script for analysis (ZIP 6783 kb)

## Abbreviations

SeeD: Seeds of Discovery
*N*_*e*_: Effective population size
*F*: Expected coefficient of inbreeding within landrace
GBS: Genotyping-by-Sequencing
GBS10x@10K: 10,000 GBS markers with 10x coverage
GBS1x@100K: 100,000 GBS markers with 1x coverage
QTL: Quantitative trait loci (QTL)
TBV: True breeding value
EBV: Estimated breeding value
*h*^*2*^: Heritability
Landrace approach: Initiate pre-bridging germplasm from the selected landraces
LandraceDH approach: Initiate pre-bridging germplasm from doubled haploids of the selected landraces
LandraceElite approach: Initiate pre-bridging germplasm from testcrosses of the selected landraces
