# Supplementary material for: Initiating maize pre-breeding programs using genomic selection to harness polygenic variation from landrace populations
Source: BMC Genomics. 2016 Jan 5;17:30. doi: 10.1186/s12864-015-2345-z (PMC4702314; doi:10.1186/s12864-015-2345-z)
Supplement: Additional file 1: — Detailed description of generating i) genomes of landraces and the elite hybrid, ii) marker genotype data, and iii) trait genetic architecture, quantitative loci, true breeding values, and phenotypes. (PDF 424 kb) [file 12864_2015_2345_MOESM1_ESM.pdf]

Initiating maize pre-breeding programs using genomic selection to harness polygenic variation from landrace populations

Supplementary methods

Gregor Gorjanc[1,2,§], Janez Jenko[2,3], Sarah J. Hearne[4], John M. Hickey[2]

[1]Biotechnical Faculty, University of Ljubljana, 1000 Ljubljana, Slovenia

[2]The Roslin Institute and Royal (Dick) School of Veterinary Studies, The University of Edinburgh, Easter Bush, Midlothian, Scotland, UK

[3]Agricultural Institute of Slovenia, 1000 Ljubljana, Slovenia

[4]Genetic Resources Program, International Maize and Wheat Improvement Center (CIMMYT), Apdo. 06600 México D.F., México

Genomes

Genome simulation started by generating the founding population with 3,000 haplotype sequences for each of 10 chromosomes using the Markovian Coalescent Simulator (MaCS) [71]. Each chromosome was 200 cM long and included 2×10^8 base pairs. Chromosomes were simulated using a per site recombination rate of 1.0×10^{-8}, per site mutation rate of 2.5×10^{-8}, and an effective population size (N_e) that varied over time in two scenarios. The first scenario mimicked the large diversity around the time of maize domestication that gave rise to a wide range of landrace populations. This was achieved by setting the effective population size to 100,000, leading to 270×10^6 segregating sites in the simulated haplotypes. The second scenario mimicked reduced diversity that would be contained by a subset of landrace populations. This was achieved by setting the effective population size to 100,000 at 6,000 years ago, to 10,000 at 2,000 years ago, and to 1,000 in the final generation of coalescent simulation with linear interpolation in between, leading to 10×10^6 segregating sites in simulated haplotypes.

The phenotyping of polygenic traits in SeeD was performed in crosses between landrace individuals and single cross elite hybrids to gauge the potential testcross performance without introducing confounding effects of heterotic pattern. To simulate an elite hybrid genome the coalescent simulation involved a population split 50 years ago and the best haplotype was selected from each of the two subpopulations based on true breeding values (see section Trait genetic architecture and phenotypes). To initiate creation of landraces the bottom half of haplotypes from one subpopulation were selected based on true breeding values. This collection of haplotypes was sampled with replacement to initiate creation of 3,000 independent landraces. The landraces were simulated in two ways to reflect different levels of differentiation among them. This was achieved by simulating landrace populations with the expected inbreeding coefficient (F) of either 0.3 or 0.9 since the start of a landrace population, which represents high or low levels of variation within landraces respectively, and consequently low or high levels of variation between landraces. The expected inbreeding coefficient of 0.3 or 0.9 was achieved by random mating (including selfing) of 5 or 1 individuals for 7 or 9 generations, respectively [72]. Finally, the last generation individuals were mated at random to generate a collection of 100 seeds per landrace.

Genotyping platform

Genotyping-by-Sequencing (GBS) technology [25] was chosen as the genotyping platform within SeeD to avoid ascertainment bias of standard single nucleotide polymorphism (SNP) arrays [e.g., 25, 73]. We addressed whether GBS marker genotypes should be called accurately for a medium number of markers or less accurately for a high number markers. It was assumed these two scenarios could be covered with GBS with 10*x* coverage at 10,000 markers (GBS10*x*@10K) or with 1*x* coverage at 100,000 markers (GBS1*x*@100K). These markers were sampled from the segregating sites of the simulated haplotypes with the

restriction that an equal number of markers were selected from each chromosome. Genotype calling from marker site sequence reads was simulated as described in [38].

Trait genetic architecture and phenotypes

The trait of interest was assumed to be an index composed of several polygenic traits, which a pre-breeding program manager would select upon. We have simulated such an index as a single trait with 20,000 bi-allelic quantitative trait loci (QTL), with each chromosome harboring an equal number of QTL. A large number of QTL was chosen due to the assumed polygenic nature of traits included in the index and our expectation that the number of causative nucleotide changes is large in a diverse population simulated in this study. The effects of QTL were simulated from Gaussian distribution with mean zero and variance of $1/n_{QTL}$, where n_{QTL} is the number of QTL. The true breeding value (**TBV**) of an individual was obtained as the sum of effects of QTL alleles an individual inherited. Phenotypes with heritability (h^2) of 0.25 or 0.50 were generated as a mean of two replicates each representing one-row plots with ten plants. Plant phenotypes were simulated from Gaussian distribution with mean equal to TBV of a plant and residual variance of $\sigma^2_{e,plant} = (\sigma^2_a/h^2 - \sigma^2_a)n_p$, where σ^2_a is the variance of breeding values in the founding population and $n_p = 20$ is the number of plants grown.
