# Supplementary material for: Initiating maize pre-breeding programs using genomic selection to harness polygenic variation from landrace populations
Source: BMC Genomics. 2016 Jan 5;17:30. doi: 10.1186/s12864-015-2345-z (PMC4702314; doi:10.1186/s12864-015-2345-z)
Supplement: Additional file 2: — Fig. {S1, S2, S3}. Scheme of the discovery phase with the {Landrace,LandraceDH,LandraceElite} approach; Fig. S4. Scheme of the improvement phase; Fig. {S5, S6, S7, S8}. {Genetic merit, Kinship with the elite hybrid, Accuracy of genomic evaluation/prediction, Heterozygosity} in different stages by approach, genetic diversity of the founding population, genetic diversity within accessions, and heritability; Fig. {S9, S10, S11}. Decision tree for accuracy of {selecting accessions in the discovery phase, selecting seeds from the selected accessions in the discovery phase, in the final stage of improvement phase}; Table {S1, S2, S3, S4}. {Genetic merit, Kinship with the elite hybrid, Accuracy of genomic evaluation/prediction, Heterozygosity} in different stages by approach, genetic diversity of the founding population, genetic diversity within accessions, and heritability. (PDF 4858 kb) [file 12864_2015_2345_MOESM2_ESM.pdf]

Initiating maize pre-breeding programs using genomic selection to harness polygenic variation from landrace populations

Supplementary figures and tables

Gregor Gorjanc[1,2,§], Janez Jenko[2,3], Sarah J. Hearne[4], John M. Hickey[2]

[1]Biotechnical Faculty, University of Ljubljana, 1000 Ljubljana, Slovenia

[2]The Roslin Institute and Royal (Dick) School of Veterinary Studies, The University of Edinburgh, Easter Bush, Midlothian, Scotland, UK

[3]Agricultural Institute of Slovenia, 1000 Ljubljana, Slovenia

[4]Genetic Resources Program, International Maize and Wheat Improvement Center (CIMMYT), Apdo. 06600 México D.F., México

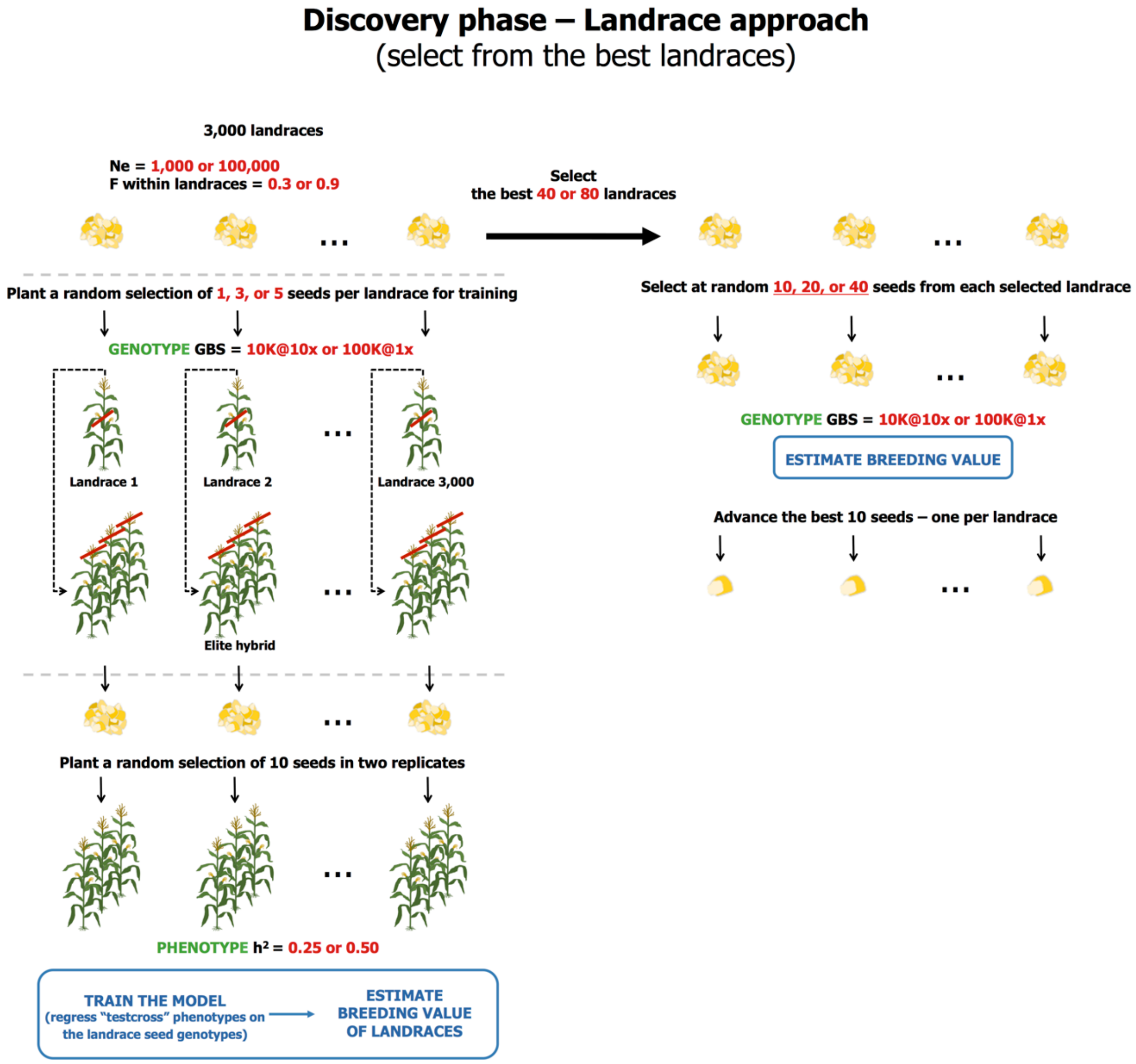

Fig. S1. Scheme of the discovery phase with the Landrace approach

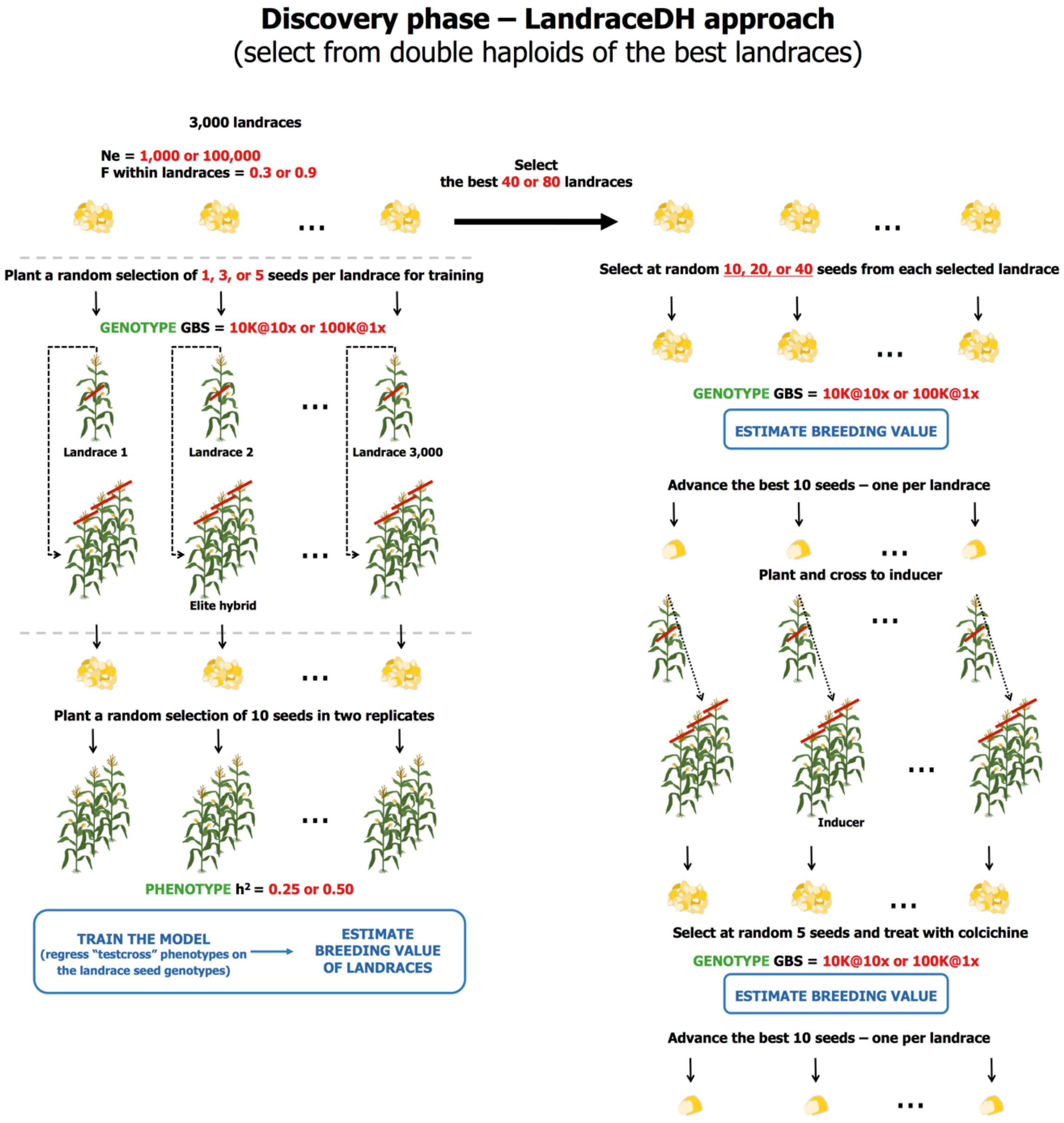

Fig. S2. Scheme of the discovery phase with the LandraceDH approach

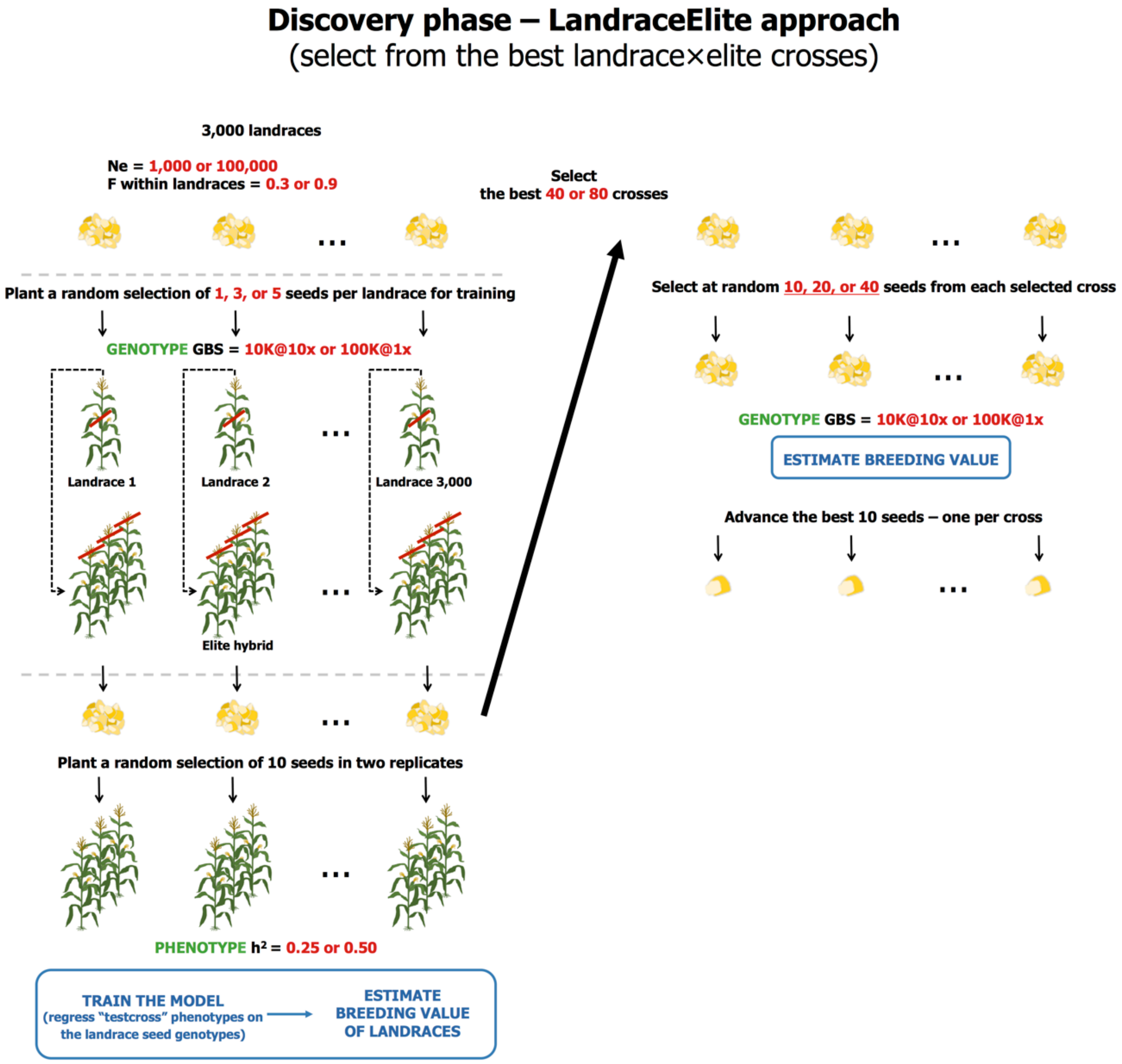

Fig. S3. Scheme of the discovery phase with the LandraceElite approach

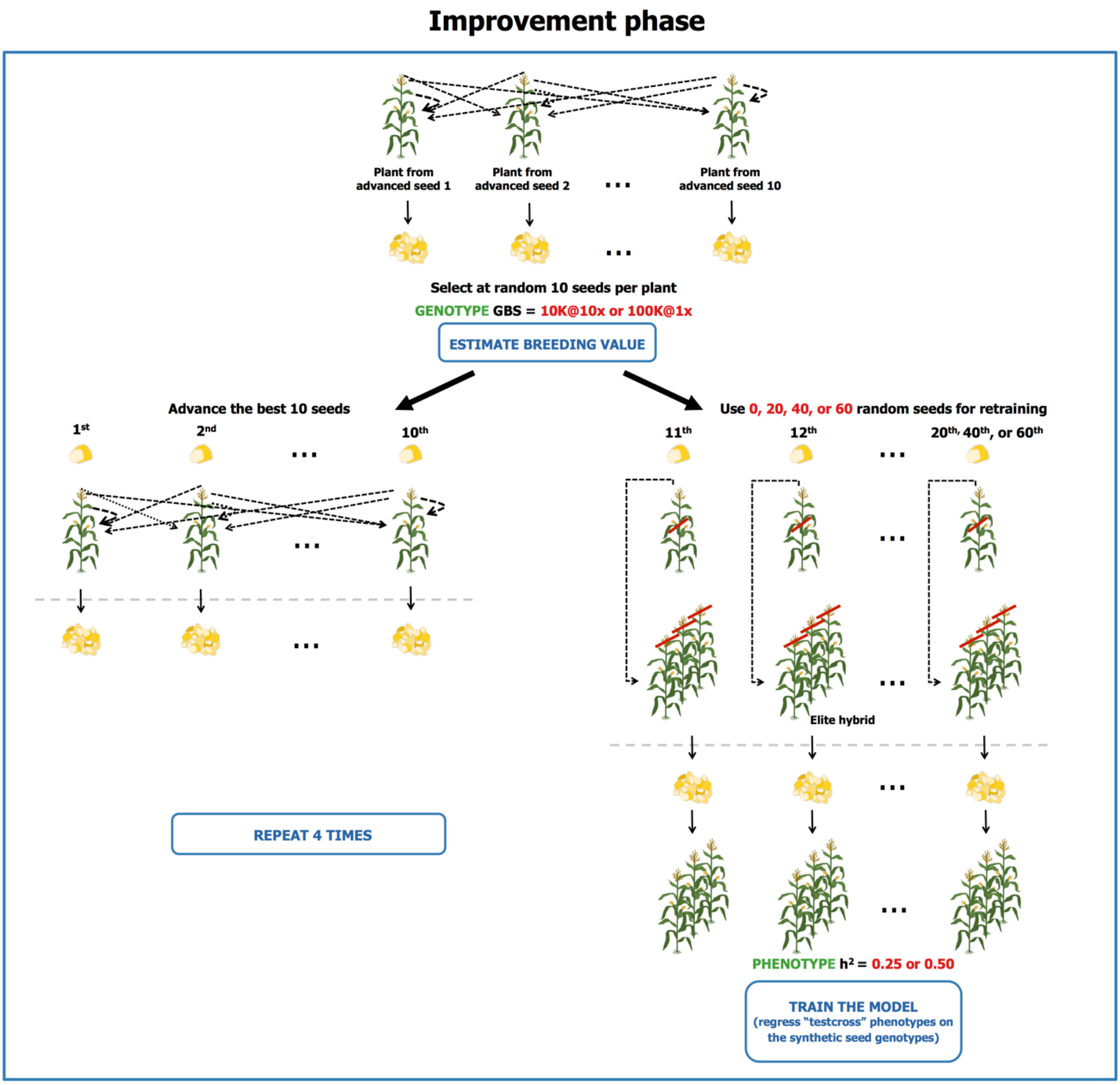

Fig. S4. Scheme of the improvement phase

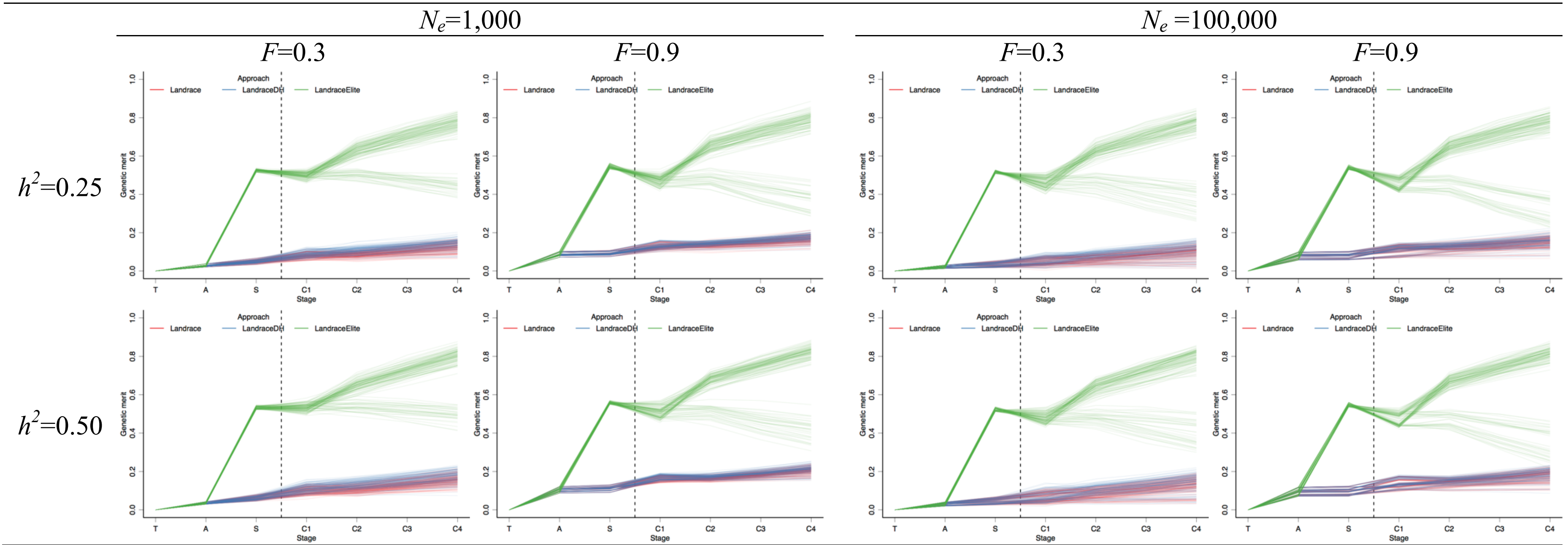

Fig. S5. Genetic merit in different stages of the discovery phase (training – T, the selected accessions – A, the selected seeds from the selected accessions – S) and the improvement phase (the selected seeds in each of the four cycles – C1-C4) by approach, genetic diversity of the founding population (N_e=1,000 or N_e=100,000), genetic diversity within accessions (F=0.3 or F=0.9), and heritability (h^2=0.25 or h^2=0.5) (one line represents a mean over ten replicates)

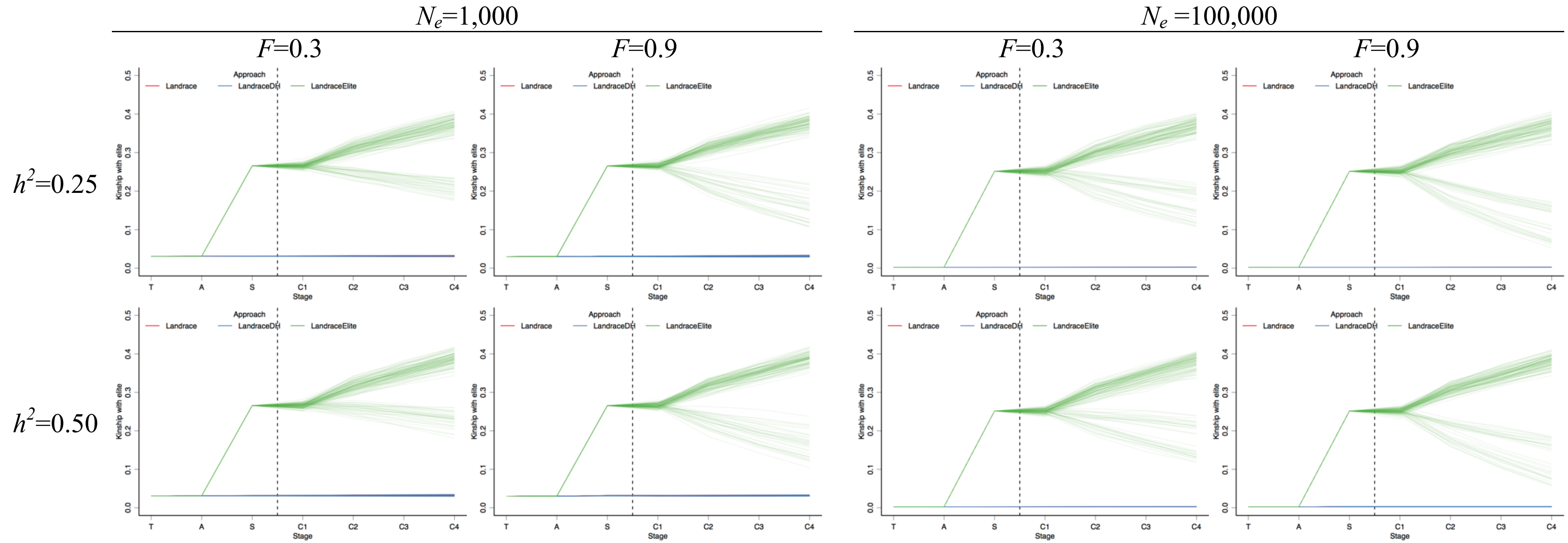

Fig. S6. Kinship with the elite hybrid in different stages of the discovery phase (training – T, the selected accessions – A, the selected seeds from the selected accessions – S) and the improvement phase (the selected seeds in each of the four cycles – C1-C4) by approach, genetic diversity of the founding population (N_e=1,000 or N_e=100,000), genetic diversity within accessions (F=0.3 or F=0.9), and heritability (h^2=0.25 or h^2=0.5) (one line represents a mean over ten replicates)

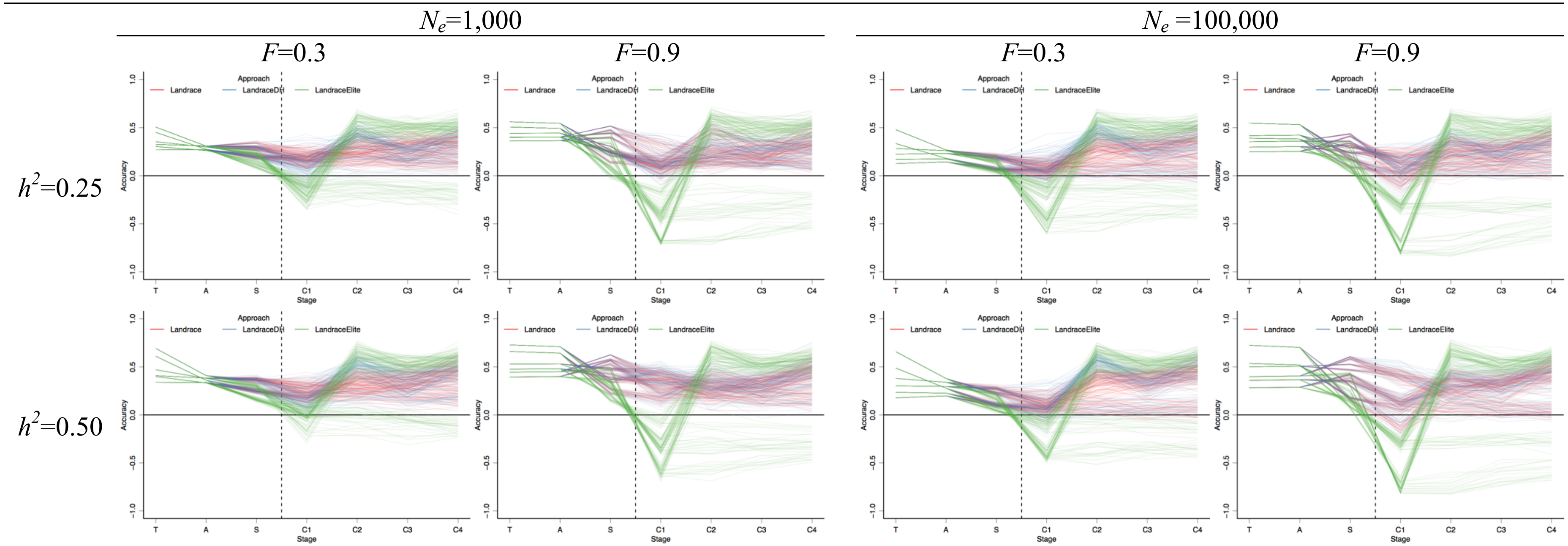

Fig. S7. Accuracy of genomic evaluation/prediction in different stages of the discovery phase (training – T, selection of accessions – A, selection of seeds from the selected accessions – S) and the improvement phase (selecting seeds in each of the four cycles – C1-C4) by approach, genetic diversity of the founding population (N_e=1,000 or N_e=100,000), genetic diversity within accessions (F=0.3 or F=0.9), and heritability (h^2=0.25 or h^2=0.5) (one line represents a mean over ten replicates)

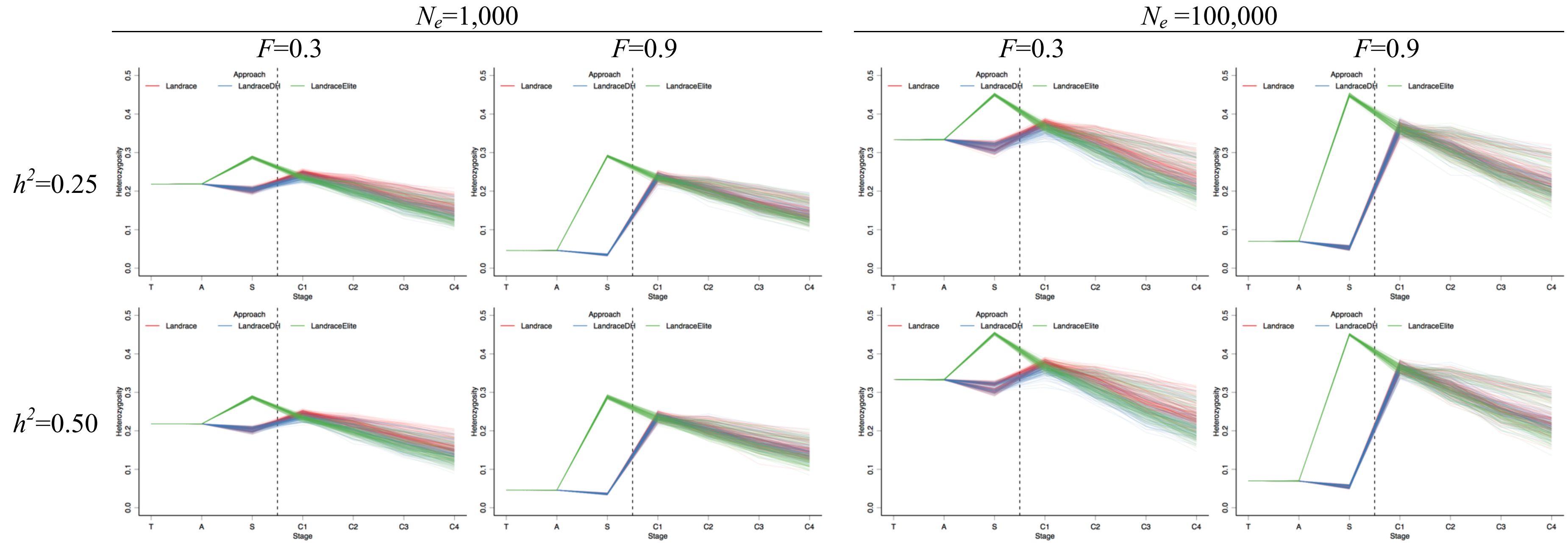

Fig S8. Heterozygosity in different stages of the discovery phase (training – T, the selected accessions – A, the selected seeds from the selected accessions – S) and the improvement phase (the selected seeds in each of the four cycles – C1-C4) by approach, genetic diversity of the founding population (N_e=1,000 or N_e=100,000), genetic diversity within accessions (F=0.3 or F=0.9), and heritability (h^2=0.25 or h^2=0.5) (one line represents a mean over ten replicates)

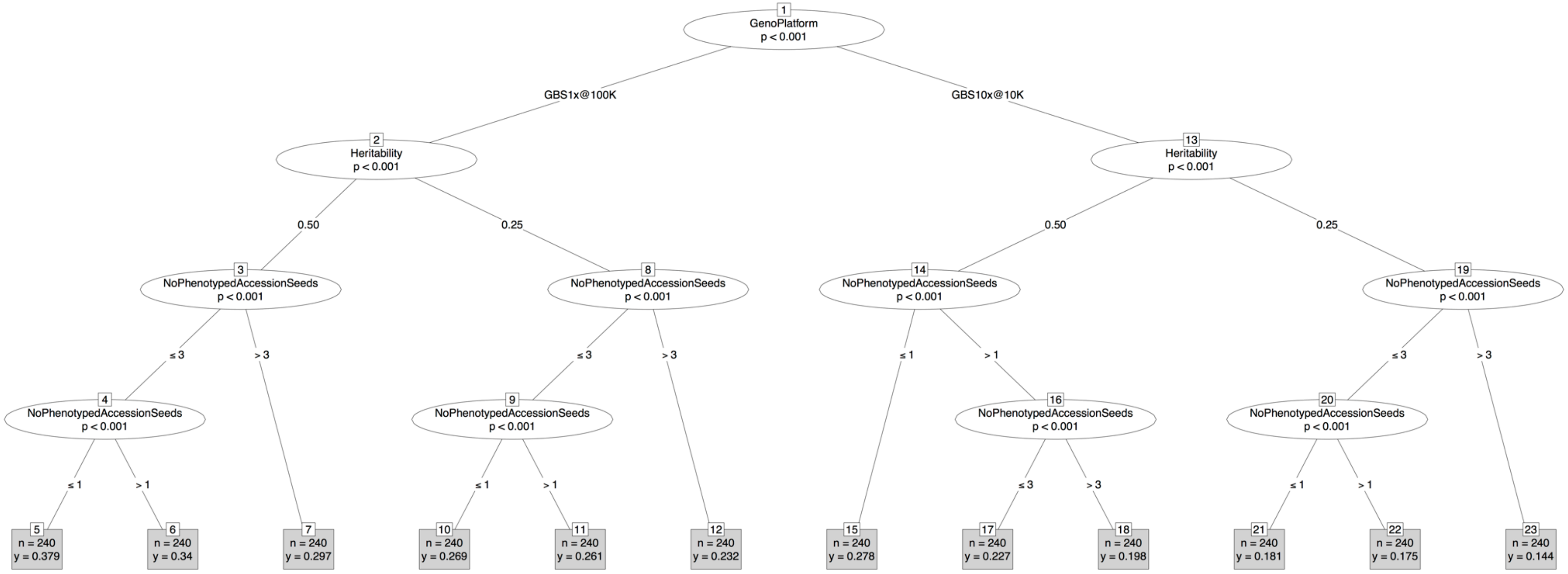

Fig. S9. Decision tree for accuracy of selecting accessions in the discovery phase with the Landrace approach, high diversity of the founding population (N_e=100,000), and high diversity within accessions (F=0.3)

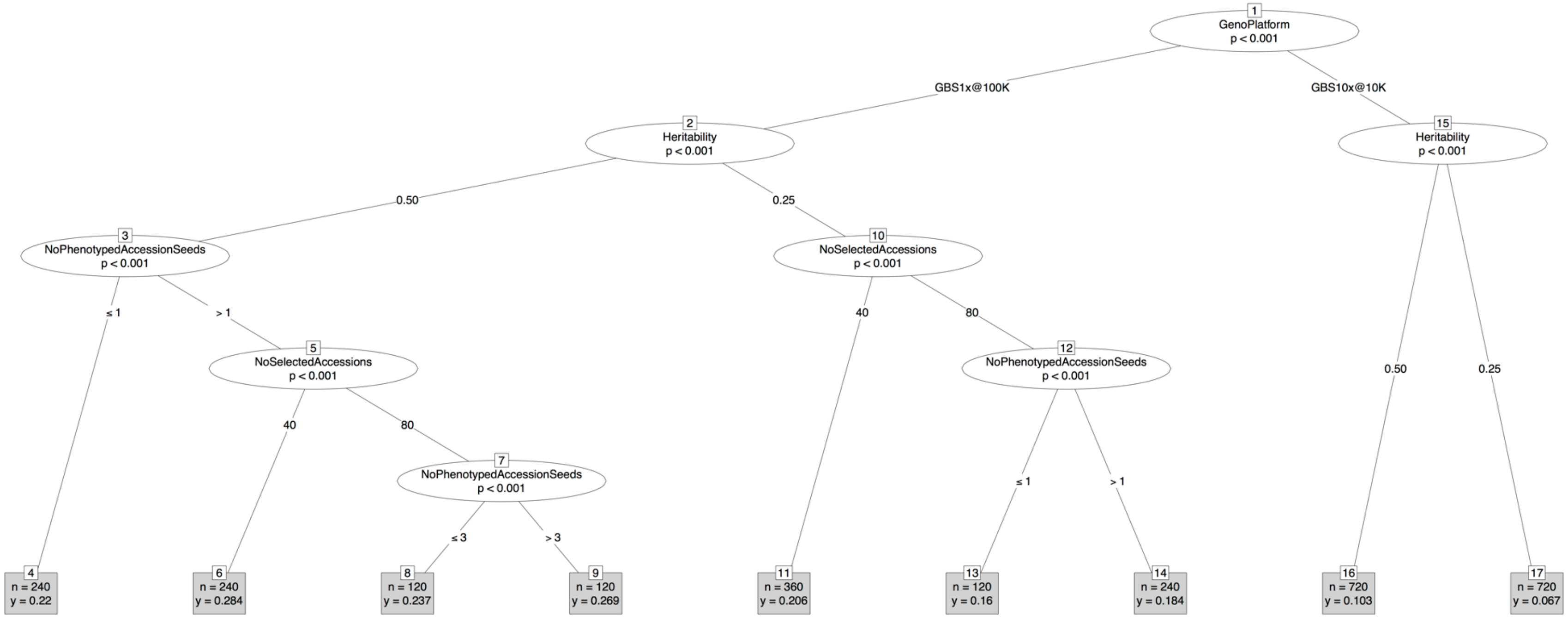

Fig. S10. Decision tree for accuracy of selecting seeds from the selected accessions in the discovery phase with the Landrace approach, high diversity of the founding population (N_e=100,000), and high diversity within accessions (F=0.3)

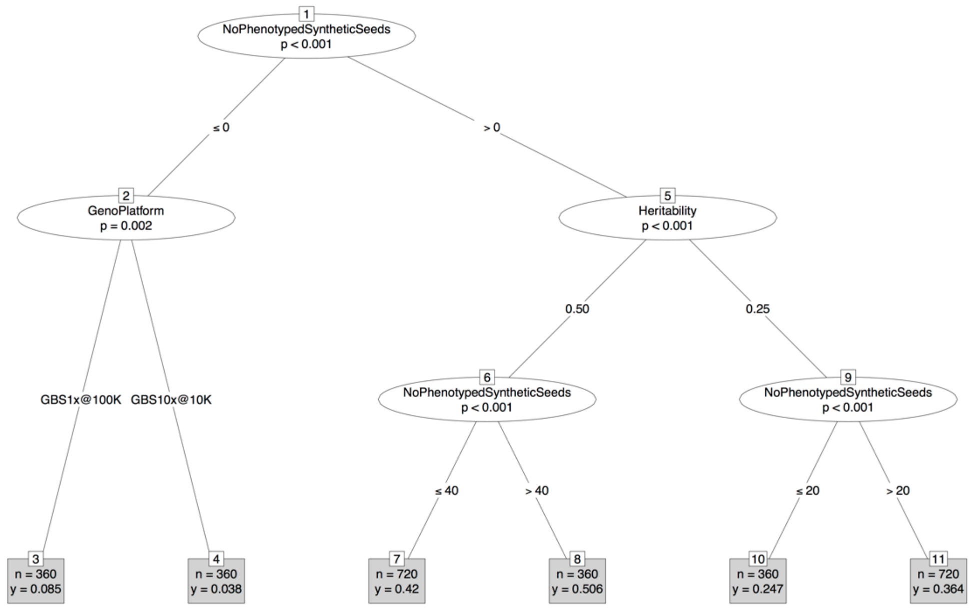

Fig. S11. Decision tree for accuracy in the final stage of improvement phase with the Landrace approach, high diversity of the founding population (N_e=100,000), and high diversity within accessions (F=0.3)

Table S1. Genetic merit (mean and 95% quantiles over scenarios and replicates) in different stages of the discovery phase (training – T, the selected accessions – A, the selected seeds from the selected accessions – S) and the improvement phase (the selected seeds in each of the four cycles – C1-C4) by approach

Approach/Stage	T	A	S	C1	C2	C3	C4
Landrace	0.00 (0.00, 0.00)[a]	0.06 (0.02, 0.12)[a]	0.07 (0.02, 0.12)[a]	0.10 (0.02, 0.19)[a]	0.11 (0.02, 0.19)[a]	0.13 (0.03, 0.22)[a]	0.15 (0.03, 0.25)[a]
LandraceDH	0.00 (0.00, 0.00)[a]	0.06 (0.02, 0.12)[a]	0.07 (0.02, 0.12)[a]	0.11 (0.01, 0.20)[b]	0.12 (0.03, 0.20)[b]	0.14 (0.03, 0.23)[b]	0.16 (0.04, 0.26)[b]
LandraceElite	0.00 (0.00, 0.00)[a]	0.06 (0.02, 0.12)[a]	0.54 (0.51, 0.57)[b]	0.48 (0.39, 0.60)[c]	0.61 (0.40, 0.76)[c]	0.65 (0.32, 0.84)[c]	0.70 (0.26, 0.92)[c]

Means with different letter within a column are different at $p<0.01$

Table S2. Kinship with the elite hybrid (mean and 95% quantiles over scenarios and replicates) in different stages of the discovery phase (training – T, the selected accessions – A, the selected seeds from the selected accessions – S) and the improvement phase (the selected seeds in each of the four cycles – C1-C4) by approach

Approach/Stage	T	A	S	C1	C2	C3	C4
Landrace	0.02 (0.00, 0.03)[a]	0.02 (0.00, 0.03)[a]	0.02 (0.00, 0.03)[a]	0.02 (0.00, 0.03)[a]	0.02 (0.00, 0.04)[a]	0.02 (0.00, 0.04)[a]	0.02 (0.00, 0.05)[a]
LandraceDH	0.02 (0.00, 0.03)[a]	0.02 (0.00, 0.03)[a]	0.02 (0.00, 0.03)[a]	0.02 (0.00, 0.03)[a]	0.02 (0.00, 0.04)[a]	0.02 (0.00, 0.04)[a]	0.02 (0.00, 0.05)[a]
LandraceElite	0.02 (0.00, 0.03)[a]	0.02 (0.00, 0.03)[a]	0.26 (0.25, 0.27)[b]	0.26 (0.22, 0.29)[b]	0.29 (0.17, 0.36)[b]	0.31 (0.12, 0.40)[b]	0.32 (0.09, 0.44)[b]

Means with different letter within a column are different at $p<0.01$

Table S3. Accuracy of genomic evaluation/prediction (mean and 95% quantiles over scenarios and replicates) in different stages of the discovery phase (training – T, selected accessions – A, selected seeds from the selected accessions – S) and the improvement phase (selected seeds in each of the four cycles – C1-C4) by approach

Approach/Stage	T	A	S	C1	C2	C3	C4
Landrace	0.42 (0.17, 0.72)[a]	0.37 (0.17, 0.70)[a]	0.28 (0.01, 0.61)[a]	0.16 (-0.30, 0.60)[a]	0.26 (-0.19, 0.67)[a]	0.25 (-0.21, 0.68)[a]	0.32 (-0.20, 0.77)[a]
LandraceDH	0.42 (0.17, 0.72)[a]	0.37 (0.17, 0.70)[a]	0.28 (0.01, 0.61)[a]	0.14 (-0.47, 0.71)[b]	0.33 (-0.17, 0.77)[b]	0.25 (-0.23, 0.69)[a]	0.33 (-0.21, 0.80)[ab]
LandraceElite	0.42 (0.17, 0.72)[a]	0.37 (0.17, 0.70)[a]	0.18 (-0.04, 0.50)[b]	-0.33 (-0.83, 0.20)[c]	0.34 (-0.74, 0.83)[b]	0.30 (-0.63, 0.78)[b]	0.33 (-0.59, 0.83)[b]

Means with different letter within a column are different at $p<0.01$

Table S4. Heterozygosity (mean and 95% quantiles over scenarios and replicates) in different stages of the discovery phase (training – T, the selected accessions – A, the selected seeds from the selected accessions – S) and the improvement phase (the selected seeds in each of the four cycles – C1-C4) by approach

Approach/Stage	T	A	S	C1	C2	C3	C4
Landrace	0.17 (0.04, 0.34)[a]	0.17 (0.04, 0.34)[a]	0.15 (0.03, 0.33)[a]	0.31 (0.21, 0.40)[a]	0.27 (0.16, 0.39)[a]	0.23 (0.11, 0.35)[a]	0.19 (0.08, 0.32)[a]
LandraceDH	0.17 (0.04, 0.34)[a]	0.17 (0.04, 0.34)[a]	0.15 (0.03, 0.33)[a]	0.30 (0.18, 0.40)[b]	0.26 (0.15, 0.39)[b]	0.22 (0.09, 0.34)[b]	0.18 (0.07, 0.31)[b]
LandraceElite	0.17 (0.04, 0.34)[a]	0.17 (0.04, 0.34)[a]	0.37 (0.28, 0.46)[b]	0.30 (0.21, 0.40)[b]	0.26 (0.16, 0.38)[c]	0.21 (0.11, 0.35)[c]	0.17 (0.07, 0.31)[c]

Means with different letter within a column are different at $p<0.01$
